# 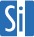
Lexical is as lexical does: computational approaches to lexical representation

**DOI:** 10.1080/23273798.2015.1005637

**Published:** 2015-02-18

**Authors:** Anna M. Woollams

**Affiliations:** ^a^ Neuroscience and Aphasia Research Unit, School of Psychological Sciences, University of Manchester, Zochonis Building, Brunswick Street, ManchesterM13 9PL, UK

**Keywords:** lexical representation, word recognition, orthography, phonology, semantics, computational models

## Abstract

In much of neuroimaging and neuropsychology, regions of the brain have been associated with ‘lexical representation’, with little consideration as to what this cognitive construct actually denotes. Within current computational models of word recognition, there are a number of different approaches to the representation of lexical knowledge. Structural lexical representations, found in original theories of word recognition, have been instantiated in modern localist models. However, such a representational scheme lacks neural plausibility in terms of economy and flexibility. Connectionist models have therefore adopted distributed representations of form and meaning. Semantic representations in connectionist models necessarily encode lexical knowledge. Yet when equipped with recurrent connections, connectionist models can also develop attractors for familiar forms that function as lexical representations. Current behavioural, neuropsychological and neuroimaging evidence shows a clear role for semantic information, but also suggests some modality- and task-specific lexical representations. A variety of connectionist architectures could implement these distributed functional representations, and further experimental and simulation work is required to discriminate between these alternatives. Future conceptualisations of lexical representations will therefore emerge from a synergy between modelling and neuroscience.

The term ‘lexical representation’ is commonly found in much recent work concerning the neural bases of normal and disordered language processing, and has a longer history in the context of experimental and theoretical psycholinguistics. Indeed, a search for this term on SCOPUS yields more than 3300 results. Yet, when this term is used, it is often without any formal definition: if one adds ‘definition’ to our search, this currently yields less than 100 results. Both the popularity of the term and the paucity of formal definition arise from the fact that most researchers in the field of language processing feel that we have an intuitive sense of what ‘lexical representation’ means. Broadly, it derives from our sense that there is some form of ‘mental lexicon’ or internal dictionary, in which the knowledge we have concerning the words we know is represented. Of course, our semantic system represents the meaning of known words, and there has been considerable debate concerning the extent to which this obviates the need for any other form of whole word representations. This paper surveys a range of possible implementations of lexical representations within current computational models of word recognition and production with particular attention to the role of semantics. In addition to semantic activation for familiar words, a proposal is put forward for distributed functional lexical representations, which incorporate a degree of modality and task specificity. Recent neuroimaging literature concerning lexical representations is then considered that supports a proposal for multiple levels of distributed functional lexical representations. This highlights the areas in which further research is needed to understand the nature of lexical representations at the cognitive and neural levels.

## Why do we need lexical representations?

The existence of some form of lexical representation is inferred when a behavioural processing advantage emerges for a familiar string of letters or phonemes (e.g., *DOG*) over a novel string (e.g., *POG*), which is termed the lexicality effect. The lexicality effect is pervasive across a variety of psycholinguistic tasks, with the key ones that have informed the development of models of written and spoken word recognition including letter/phoneme identification, visual/auditory lexical decision and reading aloud/repetition. In surveying this literature, two issues emerge as important for theoretical interpretation of the lexicality effect. The first is the extent to which the advantage for words is seen over *comparable nonwords*, namely those with subword components that are similar to those seen in words (as measured by bigraph/biphone probabilities and/or neighbourhood/cohort sizes). If a lexicality effect is seen with nonwords that are distinguishable in terms of their subword properties (e.g., *DOG* vs *ZQF*), this is not necessarily evidence for lexical representation, but rather of familiarity with subword components. The second is the extent to which lexicality effects seen with comparable nonwords may be driven by activation of *semantic representations*. If we see lexicality effects with closely matched nonwords that are always accompanied by significant effects of semantic variables such as imageability, then this suggests that in fact lexical representations could potentially be reduced to semantic information.

The classic version of the letter identification task involves brief presentation of a string of letters followed by two alternatives over a particular letter position (e.g., *DOG*, followed by a choice of G or T over the third position). In this task, there is a clear advantage for letters presented in a pseudoword relative to an unpronounceable letter string (i.e., better identification of G in *POG* vs *PZG*) (McClelland & Johnston, 1977; Paap, Newsome, McDonald, & Schvaneveldt, 1982). This pseudoword superiority effect indicates familiarity with subword components. More importantly, there is an additional advantage for letters presented in a real word relative to a pronounceable pseudoword (i.e., better identification of G in *DOG* vs *POG*) (Manelis, 1974; McClelland & Johnston, 1977). There is some evidence that this word superiority effect emerges from the familiarity of not only orthography, but also phonology, as participants can be misled when presented with pseudohomophonic nonwords, although the activation of phonology may be subject to strategic influences (Hooper & Paap, 1997). While the word superiority effect equates to a lexicality effect, to date there has been no exploration of the influence of semantic variables upon letter identification performance. In the auditory domain, there is a clear influence of lexical knowledge on perception such that when required to judge whether ‘p’ or ‘b’ is heard, this is biased by lexicality, such that the shift occurs earlier when moving from ‘beace’ to ‘peace’, and later when moving from ‘beef’ to ‘peef’ (Ganong, 1980; Miller, Dexter, & Pickard, 1984). We are also more likely to fail to notice a missing phoneme in a heard word than nonword (Samuel, 1996). Whether this effect can be reduced to semantics is unclear, as while semantic context increases the likelihood of phoneme restoration (Liederman et al., 2011), the magnitude of such effects relative to those of lexicality have yet to be directly compared.

Turning to lexical decision, where the task is simply to judge if a string is a word, there is universally a clear advantage for words over nonwords. Lexical decision is a type of signal detection task where the nature of the nonword foils (the noise) relative to the word targets (the signal) critically determines the ‘depth’ of processing needed for effective discrimination. In visual lexical decision, the lexicality effect is negligible when the nonwords are less plausible (e.g., *KZT*), and increases with pronounceable foils (e.g., *KET*) and is largest with pseudohomophones (e.g., *KAT*). At the same time, semantic effects, like imageability or semantic priming, increase as the nonwords foils become more word-like (Evans, Lambon Ralph, & Woollams, 2012; James, 1975; Joordens & Becker, 1997). Although lexicality and semantic effects in visual lexical decision emerge in parallel, the lexicality effects observed are larger than the semantic effects (Evans et al., 2012). This difference is, however, difficult to interpret, given that lexicality is inherently confounded with the response required. In auditory lexical decision, lexicality effects are also seen, and semantic effects are apparent for items from larger cohorts (Tyler, Voice, & Moss, 2000). Similar to the visual domain, it is harder to reject more word-like spoken nonwords (Vitevitch & Luce, 1999; Vitevitch, Luce, Pisoni, & Auer, 1999), although the influence of foil type upon performance for words has yet to be investigated in the auditory domain.

A clear lexicality effect is also seen when a spoken response is required, as in reading aloud (McCann & Besner, 1987) and repetition (Vitevitch & Luce, 1998). In repetition, there is an advantage for words over nonwords, and this diminishes the more wordlike the nonword (Vitevitch & Luce, 2005). There is also evidence of semantic effects in repetition (Tyler et al., 2000; Wurm, Vakoch, & Seaman, 2004), although these have not been directly compared to those of lexicality. In reading aloud, there is an issue around the consistency of the spelling to sound correspondences, both for words and nonwords. For words, performance is slower for items containing atypical correspondences, particularly when these items are low in frequency (Jared, 1997, 2002). For nonwords, the same effect can be seen, such that items containing inconsistent correspondences are slower (Andrews & Scarratt, 1998). Hence, the lexicality effect in this task is largest when comparing words with typical correspondences to nonwords containing inconsistently pronounced elements. The issue of consistency is also relevant for the presence of semantic effects in this task, with these being strongest for words with atypical correspondences (Shibahara, Zorzi, Hill, Wydell, & Butterworth, 2003; Strain, Patterson, & Seidenberg, 1995, 2002; Woollams, 2005). However, there has yet to be a direct comparison between the size of the semantic effects seen in naming and the lexicality effect obtained with comparable nonwords. It is worth noting that the lexicality effect reflects familiarity of both form and meaning, whereas most semantic manipulations concern variations amongst meanings, and hence may be expected to be weaker.

Evidence from psycholinguistics indicates that the lexicality *effect* is a basic phenomenon that all models of visual and auditory word recognition must accommodate. To the extent that this cannot be reduced to a semantic contribution, then the existence of some form of lexical *representation* is therefore necessary in any model. The nature of such representations, as will become apparent, can vary from model to model across a variety of dimensions.

## What do lexical representations look like?

**Figure 1.**
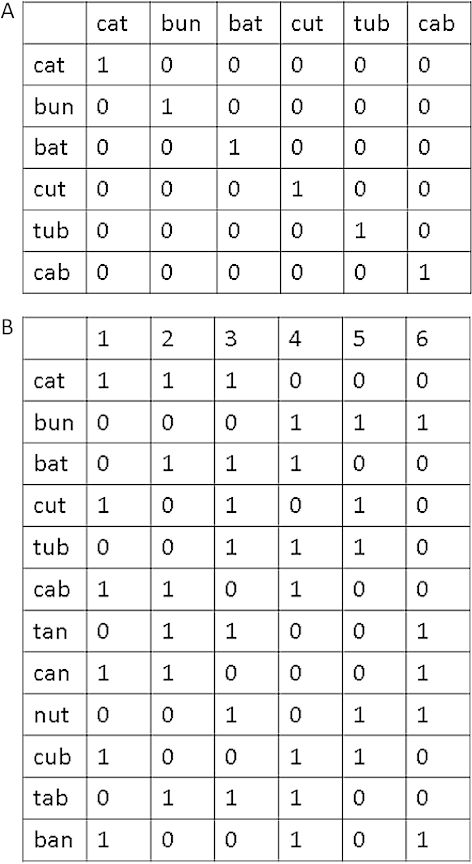
A schematic representation of activation of units encoding (a) localist representations and (b) distributed representations. In (a) representation of six words requires six units. In (b) representation of twelve words also requires six units. In (b) the representations are distributed at the word level but localist at the letter level for the purposes of exposition. A fully distributed scheme would have the capacity to represent many more words.

Models of word recognition have adopted two broad approaches to the implementation of lexical representations: a localist structural view and a distributed functional view. Models that adopt localist representations have one or more sets of units within which there is a unit corresponding to each known word (see Figure 1a). These units are dedicated to the representation of words only, and cannot support accurate processing of novel strings. The lexical units are fixed, in the sense that they exist irrespective of the current stimulus or task requirements. As there is an identity relationship between lexical items and the representational units in the model, then words can be seen as existing as part of the structure of the model.

The localist structural approach has its origins in the earliest modern conceptions of lexical representations, outlined by Morton (1969) in his proposal of the logogen model. Within this verbal theory, the written form of each known word is represented by a unit, or logogen. Each logogen can hold activation for an entry generated by presentation of a letter string, with a higher level of activation the closer the match between the internal and external representation of that string. The resting level of a particular logogen is higher the greater our familiarity with that word, and recognition occurs when activation of a particular logogen exceeds threshold, which explains the advantage for high over low-frequency words (Forster & Chambers, 1973).

The logogen model formed the basis for one of the first implemented computational models of visual word recognition, the Interactive Activation and Competition (IAC) model of Rumelhart and McClelland (1982). In this model, letter features activate letter representations, and in turn these activate localist structural lexical representations. Crucially, this model included full bidirectional connectivity between the word and letter levels, as well as within level inhibitory connections. This allowed the model to account for the lexicality effect in letter perception. An analogous approach to auditory word recognition can be seen in the TRACE model (McClelland & Elman, 1986), which can explain the influence of lexical knowledge on perception of ambiguous phonemes.

The IAC model then provided a foundation for the implementation of the lexical pathway of the Dual Route Cascaded model of visual word recognition and reading aloud (Coltheart, Rastle, Perry, Langdon, & Ziegler, 2001). This model went beyond previous implementations as it also aimed to describe the process of reading aloud in terms of co-operation between whole word lexical processing and subword rule–based grapheme-to-phoneme conversion. The model, therefore, included not only localist structural orthographic lexical representations in the form of the orthographic input lexicon, but also localist structural phonological representations in the form of the phonological output lexicon, both of which are independent from semantic representation. These two elements are also seen in other models of visual word recognition and reading aloud (e.g., MROM (Grainger & Jacobs, 1996); CDP+ and CDP++ (Perry, Ziegler, & Zorzi, 2007, 2010)). In the auditory domain, localist models have tended to focus more on either perception (e.g., MERGE (Norris, McQueen, & Cutler, 2000)) or production (e.g., WEAVER (Levelt, Roelofs, & Meyer, 1999)).

The localist structural lexical representation approach is intuitively appealing and computationally relatively easy to implement, and models using this approach have accounted for a great deal of behavioural data concerning word recognition (Coltheart et al., 2001; Levelt et al., 1999; Perry et al., 2007). Yet, one of the major disadvantages of the localist approach of a dedicated structural unit of representation for every word is that it is by no means an efficient system of representation when scaled up to the vocabulary levels of a skilled adult (around 75,000 words (Sibley, Kello, Plaut, & Elman, 2008)). This obviously has consequences when thinking about how such a system may be implemented in the brain, which, although it has billions of neurons, nevertheless needs to accommodate many other functions apart from language processing.

In an attempt to address these concerns, Seidenberg and McClelland (1989) introduced a new approach to the modelling of visual word recognition and reading aloud. Within this model, orthographic and phonological representations consisted of subword units more akin to groups of neurons. These representations are distributed in the sense that the activation of multiple structural units contributes to the representation of a single written or spoken word, and these same units can also represent not only other words but also nonwords. Knowledge concerning words is not stored in the units of the model, but rather on the learnt weights on the connections between them. In this context, lexical representations for a given word are constructed each time they are presented, and hence are functional rather than structural – they do not have a direct relationship to the representational units of the model. This kind of system is more efficient in the sense that a given unit can participate in the representation of multiple different words (see Figure 1b) – more than 6000 units required to represent the written or spoken forms of all monosyllabic words in English can be reduced to 111 orthographic units or 200 phonetic feature units (Harm & Seidenberg, 2004). A similar shift to functional distributed representations of phonology and semantics can be seen in the domain of spoken word recognition (Gaskell & Marslen-Wilson, 1997) and production (Harm & Seidenberg, 2004).

The Seidenberg and McClelland (1989) model represented a major shift in thinking about reading, and its ability to perform lexical decision was scrutinised closely (Besner, Twilley, Seergobin, & McCann, 1990; Fera & Besner, 1992; Seidenberg & McClelland, 1990). Lexical decision is rapidly and accurately achieved by skilled readers and considered to be a basic function that any model of visual word recognition must be able to capture. As distributed functional representations involve units that can also represent nonwords, this was taken by some as problematic for lexical decision. While the initial model was able to discriminate between words and nonwords on the basis of their subword orthographic and/or phonological familiarity when the nonwords were less familiar, form-based representations were not sufficient in the case where the nonword foils were closely matched to the words (Seidenberg & McClelland, 1990).

In models using distributed functional representations, when the task is to discriminate words from very word-like nonwords, recourse has to be made to semantic information. For example, Plaut's (1997) model included an opaque set of semantic representations (in that each unit did not correspond to an underlying feature). Harm and Seidenberg's (2004) model included a transparent set of semantic representations (where each unit did correspond to an underlying feature), as did Chang, Lambon Ralph, Furber, and Welbourne's (2013) model. All of these models were able to discriminate between words (e.g., *BRAIN*) and closely matched nonwords that are homophonic with real words (i.e., pseudohomophones like *BRANE*) effectively, albeit via different metrics. In Plaut's (1997) model, discrimination was based on semantic stress, which reflects how easily a pattern of activation settles. In Harm and Seidenberg's (2004) model, the discrimination was based on how closely the internally generated orthography matched the stimulus. In Chang et al.'s (2013) model, the decision was made on the basis of pooled activation over orthographic, phonological and semantic units. This latter model has also simulated the larger semantic effects are seen in lexical decision performance for words when presented in a difficult relative to an easy foil context. In these models, therefore, semantic information clearly contributes to the representation of lexical items.

## Lexical versus semantic representations

The emergence of an alternative to localist structural representations in the form of distributed functional representations led to considerable debate in the literature concerning the existence of structural lexicons in the mind and brain (e.g., Coltheart, 2004; Elman, 2004). As described earlier, while there is evidence of the involvement of semantic information in tasks demonstrating lexicality effects, it is only neuropsychological evidence that speaks to the necessity of such information in visual and auditory word recognition. The localist structural view invokes one or more form-based lexica that are dedicated to the representation of words, irrespective of semantics. In contrast, the distributed functional view invokes activation of semantic information to support word recognition in the absence of a structural form–based lexicon. This would seem parsimonious given that the ultimate goal of language processing is of course to communicate meaning.

Localist versus distributed approaches offer contrasting perspectives on lexical representation that make rather different predictions concerning the consequences of semantic damage for word recognition. According to the localist structural account, accurate lexical decision should still be possible in the face of severe semantic deficits, as lexical and semantic representations are independent. Evidence for this view is provided by preserved performance in visual and auditory lexical decision tasks in some cases of neuropsychological patients with impaired access to word meaning (Blazely, Coltheart, & Casey, 2005; Bormann & Weiller, 2012). Cases of co-occurring deficits in word recognition and semantic knowledge are considered to be due to the contiguity of semantic and lexical processing areas in the brain (Noble, Glosser, & Grossman, 2000).

The distributed functional approach predicts that semantic impairments will undermine lexical decision performance, but only if the foils are equally familiar to the words in terms of their orthographic and phonological components. Indeed, patients with semantic dementia show very impaired recognition of words when presented with close nonword foils, but accurate performance with distant nonword foils (Patterson et al., 2006; Rogers, Lambon Ralph, Hodges, & Patterson, 2004). This view proposes that the cases of semantic impairment with preserved lexical decision performance arise from the use of distractors that can be discriminated from words on the basis of their orthographic or phonological properties (Woollams, Ralph, Plaut, & Patterson, 2007).

It is worth noting that the implementation of semantic representations does vary over models employing distributed functional representations. Most models dealing with word recognition are trained to instantiate target semantic patterns, and in this sense, meaning functions as an output layer (Chang et al., 2013; Gaskell & Marslen-Wilson, 1997; Harm & Seidenberg, 2004; Plaut, 1997). In other models considering multiple modalities beyond word recognition, then the hidden units that mediate between the external input/output layers are allowed to develop their own representational structure (Dilkina, McClelland, & Plaut, 2008, 2010; Rogers, Lambon Ralph, Garrard, et al., 2004). In either case, amodal semantic representations can function as lexical representations. However, this is not the only possible location for distributed representations that can index word knowledge within connectionist models.

## Lexical representations as attractors

Connectionist models of word recognition have usually considered inputs and outputs as some form of orthographic or phonological representations (Dilkina et al., 2010; Gaskell & Marslen-Wilson, 1997; Harm & Seidenberg, 2004; Plaut, McClelland, Seidenberg, & Patterson, 1996). As such, these do not encode the visual or auditory signal per se, but our judgement as to a representational system that captures the salient aspects of the domain (e.g., letter or phonetic features). When semantic and phonological representations are trained in the presence of recurrent connections, attractors form that represent known patterns (Plaut et al., 1996; Plaut & Shallice, 1993). Recurrence involves feedback connections between levels of representation, and/or connections between units within a layer (this latter can also be accomplished via connections to a smaller set of clean-up units, e.g. Plaut & Shallice, 1993). The point of recurrence is that it allows the activation of a unit to be affected by other units in a continuous fashion over time. This means that there is a variety of initial activation values that will come to converge on the same final pattern. The initial range of activation values forms the attractor basin for a particular item.

The utility of attractor networks in the simulation of normal and impaired reading has been demonstrated at the level of semantics (Plaut & Shallice, 1993) and at the level of phonology (Plaut et al., 1996, Simulation 3). This latter simulation demonstrated that although semantics seems to naturally encode whole word knowledge in connectionist networks, functional lexical representations can develop in networks with a direct mapping between orthographic and phonological forms. The formation of such attractors does not imply that novel patterns cannot be represented effectively, but rather that words, as familiar patterns, will have their distributed functional representation instantiated more easily over time, as initial approximate patterns of activation can fall into the attractor, whereas the initial patterns for novel strings must avoid doing so. Attractor networks therefore naturally reproduce a processing advantage for words over nonwords.

It is possible that such attractors could be harnessed to inform word recognition and support performance in tasks like lexical decision independently of semantic activation, although this possibility has been under-explored to date. This is because recurrence at the input level risks drowning out the original signal – if pre-existing internal knowledge is allowed to influence activation very early via top–down connections, then perception will be biased towards familiar items and may become inaccurate as processing progresses (e.g., novel strings come to be seen as familiar words). As most connectionist models start with input layers of orthographic or phonological representation, then recurrent connections are not included and attractors will not form. This is not, however, because there is an assumption that this is in fact the raw input to the word recognition process, but rather because the challenges associated with modelling perception of raw inputs have been so great that it was more tractable to start with higher-level representations.

**Figure 2.**
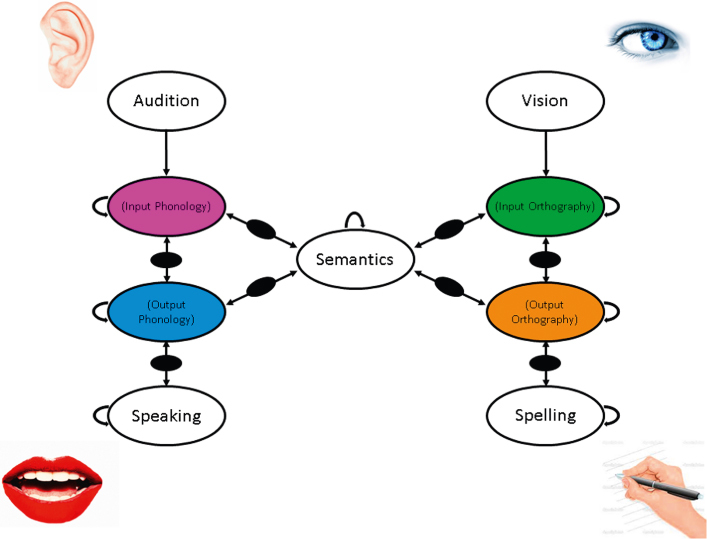
A schematic diagram of a model of visual and auditory word recognition and production showing the location of hidden unit layers that could house distributed functional lexical representations in the form of attractors. Note bidirectional connections in all cases bar those from input. Additional hidden layers are shown in black. Within level connections are shown with U-shaped arrows.

More recently, models using distributed functional representations have begun to consider mapping from raw visual inputs to meaning (Chang et al., 2013; Plaut & Behrmann, 2011), or from the acoustic signal to meaning to articulatory features (Kello & Plaut, 2004; Plaut & Kello, 1999). In these kinds of models, what we think of as orthographic or phonological lexical representations are more likely to be found in the activations of the hidden units that map between the physical inputs to outputs, either directly or via meaning (a similar idea has been eloquently articulated by Elman (2004)). The intermediate location of the orthographic/phonological representations in such models allows for recurrent connections and the formation of attractors as these are no longer direct input layers. A depiction of this kind of model is given in Figure 2. In addition, the structure of such representations will be fully distributed, in that the units no longer need to correspond to letters or phonetic features, which is even more economical. Although it has been suggested that the notion of lexical representation as hidden unit activations lacks explanatory capacity (Green, 1998), the structure of such units can be revealed using techniques such as cluster analysis (e.g., Chang, Furber, & Welbourne, 2012; Rogers, Lambon Ralph, Garrard, et al., 2004).

## Specificity of lexical representations

The concept of a lexical representation is an internal one, in that it implies at some level a degree of abstraction from physical inputs and outputs. The idea that connectionist models could contain a form of ‘lexical’ representation in the hidden units that map between different input and output domains has the advantage that it allows for a degree of specificity in terms of modality (written/spoken) and task (recognition/production), as shown in Figure 2. The need for such specificity has been suggested by the study of neuropsychological patients. With respect to modality there have been a number of reports of brain-damaged patients with problems with reading yet intact speech comprehension associated with damage to regions of the left ventral occipito-temporal cortex (vOTC) (Roberts, Lambon Ralph, & Woollams, 2010; Roberts et al., 2013; Woollams, Hoffman, Roberts, Lambon Ralph, & Patterson, 2014). Conversely, there are reports of patients with clear deficits in speech comprehension as a result of perisylvian lesions who nevertheless demonstrate relatively proficient reading comprehension (Ellis, Miller, & Sin, 1983; Lytton & Brust, 1989).

There is also some evidence of specialisation according to task requirements for comprehension or production. One of the most striking demonstrations is seen in pure alexia, where patients with profound reading difficulties are nevertheless able to write proficiently (Turkeltaub et al., 2014). This contrasts with cases of pure dysgraphia, where spelling is compromised but reading ability is retained (Miceli, Silveri, & Caramazza, 1985). Turning to spoken language processing, rare patients with word deafness due to bilateral posterior perisylvian damage are unable to understand single words but manage to speak fluently (Best & Howard, 1994). Conversely, many nonfluent patients with profound speech difficulties due to damage to Broca's area can still show good speech comprehension at the single word level (Hickok, Costanzo, Capasso, & Miceli, 2011). It should be noted, however, that it is not yet clear to what extent these modality-specific problems are due to disruption of internal orthographic/phonological representations or rather to deficits in more basic visual and auditory processing.

**Figure 3.**
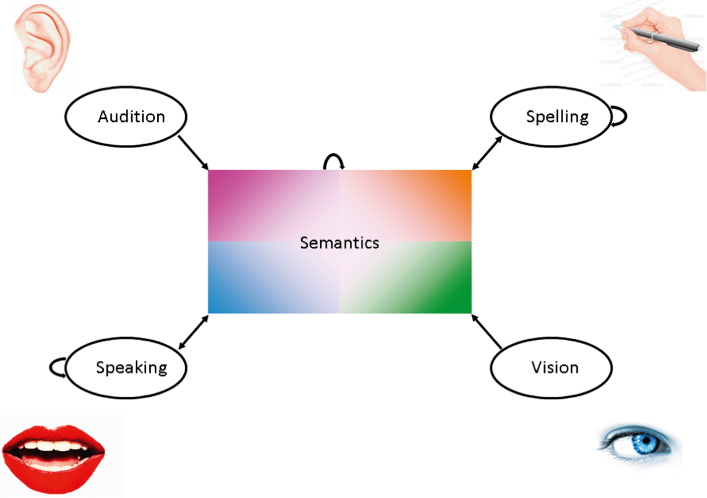
A version of the previous model of visual and auditory word recognition and production containing one large set of hidden units. Learning in the network occurs under a topographic bias that favours short connections. This allows graded modality specificity to emerge in the network, such that units close to a particular input or output participate more in tasks involving them, while units close to the centre are increasingly amodal.

Modality and task specificity of hidden unit representations can be handled in a number of different architectures in connectionist models, with the most widely used to date being the separation of hidden units onto different processing pathways (e.g., Harm & Seidenberg, 2004; Chang et al., 2013), as shown in Figure 2. A different solution in which modality specificity was captured across a set of hidden units linked their inputs or outputs was adopted by Plaut (2002), and this framework was extended to word recognition by Dilkina et al. (2010). Plaut's (2002) model set out to simulate performance in optic aphasia, where a patient is unable to name an item when presented visually, but can do so on the basis of other inputs, such as touch. Within this model, the proximity of the hidden units in two-dimensional space to a particular set of inputs or outputs captured their degree of specialisation in a graded rather than categorical way. The graded specialisation emerges because the weights on the connections of input to hidden units are biased to become stronger for closer units. When the hidden units close to visual inputs and phonological outputs were lesioned, the modality-specific naming deficit seen in optic aphasia emerged. A version of this approach applied to visual and auditory word recognition is presented in Figure 3. An interesting aspect of this approach is that as lesions to the model move towards the middle of the hidden unit space, the more domain general (i.e., semantic) their effects become. Although the spatial location of the hidden units in such models is not intended to map directly on to neural structure (Plaut, 2002), these models are interesting in that the graded specialisation according to modality and task seems to map well on to recent neuroimaging evidence concerning visual and auditory word recognition (e.g., Binney, Parker, & Lambon Ralph, 2012; Vinckier et al., 2007).

## Localisation of lexical representations

Although connectionist approaches with distributed representations were initially motivated by a desire to seek a model that is more neurally plausible than those described previously, it is only in more recent times that neural data has been used explicitly as an additional constraint in model construction and evaluation (Dell, Schwartz, Nozari, Faseyitan, & Branch Coslett, 2013; Laszlo & Plaut, 2012; Ueno, Saito, Rogers, & Lambon Ralph, 2011). Not all agree that information concerning neural structure and function are informative in terms of understanding the cognitive mechanisms that permit written and spoken word recognition and production (Coltheart, 2006, 2013). If, however, ‘the fact of the matter lies in the brain’ (Davis, 2004), then neuroimaging data provides a valuable source of information with which to limit the space of potential computational implementations.

The notion of distributed functional representations that are to some extent modality and task specific predicts that there will be more than one area of the brain that will show sensitivity to lexicality. Recent meta-analyses of both written (Cattinelli, Borghese, Gallucci, & Paulesu, 2013; Taylor, Rastle, & Davis, 2013) and spoken word recognition and production (Gow, 2012; Vigneau et al., 2006) have converged on a framework in which direct mappings between form representations (i.e., reading aloud/repetition) are attributed to a dorsal pathway while semantically mediated mappings used for word recognition are associated with a largely ventral pathway. In all of these reviews, lexicality effects are observed in multiple areas along *both* pathways. The subword direct mappings between input and outputs are flagged by greater activation for nonwords than words. In contrast, the whole word semantically mediated mappings are indicated by greater activation for words than nonwords.

It is in the areas more responsive to words than nonwords that some form of lexical representation would seem indicated. Interestingly, posterior parietal regions including the angular gyrus that show lexicality effects overlap with areas implicated in both semantic and phonological processing, and it has been suggested that this area acts as a ‘phonological lexicon’ (Davis & Gaskell, 2009; Gow, 2012; Taylor et al., 2013). Similarly, the inferior temporal regions including the fusiform gyrus that show lexicality effects have been implicated in both semantic and orthographic processing, suggesting that it functions as an ‘orthographic lexicon’ (Cattinelli et al., 2013; Davis & Gaskell, 2009; Gow, 2012; Taylor et al., 2013). These lexicality effects hold even when only studies that have carefully matched their nonwords to the words are included (Taylor et al., 2013). The interpretation of the effects in these areas is not straightforward, however. They may indicate form-based lexical representations independent of semantic activation, or the intersection of form-based and semantic processing. Both of these are consistent with the proposal of lexical representations as attractors (Chang et al., 2013; Plaut, 1996), although they vary in the units across which these would form (form-based representation vs. hidden units closer to semantics). But there is another possibility, which is that these areas are only showing higher activation for words due to feedback from higher-level semantic representations, which is consistent with the idea that lexical representations equate to semantic representations.

The use of more parametric or graded approaches to the design of neuroimaging studies on written and spoken word recognition can be informative in teasing apart alternative interpretations of the lexicality effect. An elegant study by Vinckier et al. (2007) revealed that with the progression of activation from raw visual input along the ventral pathway to the most anterior portions of the temporal lobe that contain amodal semantic representations, there is increasing sensitivity to larger orthographic clusters from single letters, to bigrams, to quadrigrams. Similarly, work by Hauk, Davies et al. (2006) has used an item-based regression approach to identify areas associated with different dimensions of form through to meaning during visual lexical decision (see also Graves, Desai, Humphries, Seidenberg, & Binder, 2010 for reading aloud). The results revealed sensitivity to more form-based variables like length and typicality for both words and nonwords in posterior brain areas, moving through to higher-level intermediate form-based representations that indexed lexicality and frequency, with more anterior areas reflecting an influence of semantic coherence (the degree to which meaning is consistent across morphologically related words). These imaging studies indicate a gradient of representation from more posterior form based to more anterior meaning based along the temporal lobe in visual word recognition. Sensitivity to lexicality in mid to posterior regions does not seem to reduce to semantic feedback as these areas are not sensitive to semantic dimensions.

As proposed earlier, in connectionist models, what we think of as orthographic or phonological lexical representations may well be found in the activations of the hidden units that map between physical inputs and outputs. Within models that incorporate a single set of hidden units and encode their modality and task specificity in terms of proximity between inputs and outputs (e.g., Dilkina et al., 2008; Plaut, 2002), the gradation from modality-specific form-based representation at the edges of the space to progressively more amodal semantic representations at the centre of the space agrees with recent conceptions of the ventral pathway in visual and auditory word recognition (see Figure 4 from Binney et al., 2012). Linking the previous proposal concerning lexical representations in hidden units to neuroimaging, along the ventral pathway, ‘lexical representations’ would be located at the intersection of form and meaning-based information. They can be localised by looking for activation that permits reliable discrimination between words and nonwords in a particular task, with newer approaches such as searchlight analysis of patterns of activation over multiple voxels seeming particularly promising in this regard (e.g., Nestor, Behrmann, & Plaut, 2013). The more similar the nonwords to words in any given task, the closer activation should move to areas involved in representation of meaning.

Not only would the precise location of lexical representation depend on stimulus properties, it would also be affected by task requirements (e.g., Binder, Medler, Westbury, Liebenthal, & Buchanan, 2006; Chen, Davis, Pulvermüller, & Hauk, 2013; Gan, Büchel, & Isel, 2013; Twomey, Kawabata Duncan, Price, & Devlin, 2011). For example, the direction of the lexicality effect observed in vOTC depends upon whether the task involves passive viewing or active discrimination (e.g., Vinckier et al., 2007; cf. Woollams, Silani, Okada, Patterson, & Price, 2011), and its presence appears to depend on the opportunity for phonological recoding (Mano et al., 2013). Perhaps even more strikingly, differing patterns of lexicality effects are observed across multiple brain regions in matching versus reading tasks for exactly the same stimuli (Vogel, Petersen, & Schlaggar, 2013). Moreover, while we see higher activation for words than nonwords in areas that process orthographic and phonological input, there is also a network of left frontal regions, which show less activation for easy-to-pronounce words than hard-to-pronounce words and nonwords (Cattinelli et al., 2013; Taylor et al., 2013). In this case, the lexicality effect occurring in speech production regions shows less effort associated with familiar word stimuli. The mediating effects of stimulus and task on the location of the brain areas specifically sensitive to the lexicality of orthographic or phonological strings emphasises the fluidity of lexical representations, as expected according to a distributed functional view.

## Activation of lexical representations

Connectionist models of word recognition are highly interactive, and feedback from higher to lower levels is integral in terms of the formation and operation of these models. As noted earlier, recurrent connections are essential for the formation of the attractors that this paper nominates as a potential candidate for functional lexical representations. From IAC and TRACE onwards, the majority of models of word recognition permit some degree of feedback activation from whole to subword information (although this is not carried as far as the input layer). A commitment to feedback is also reflected in current neural models of language processing (e.g., Price & Devlin, 2011; Ueno et al., 2011). However, others have strongly challenged the necessity of feedback connections in cognitive models of word recognition (e.g., McQueen, Cutler, & Norris, 2003; Norris et al., 2000) and also neural models (Davis, Ford, Kherif, & Johnsrude, 2011). It is very difficult to conclusively establish a role for feedback in word recognition in behavioural studies, as the response represents a summation of activation over the course of processing, and is therefore subject to ‘post-lexical’ effects. The same problem arises in studies using PET or fMRI, as again, the brain activation observed is a summation of processes over time. This leads to interpretative difficulties when dealing with lexicality effects in areas associated with form-based processing, as it is unclear to what extent these may be reduced to feedback from higher-level semantic representation.

However, neuroimaging modalities that offer good temporal resolution, such as EEG or MEG, can offer insight into the flow of brain activation during word recognition and production (for reviews see: Carreiras, Armstrong, Perea, & Frost, 2014; Mattys, Davis, Bradlow, & Scott, 2012). This can allow an understanding of the time course of the component processes involved in a behaviour, such as picture naming (Indefrey, 2011), and can also speak to the interactivity between these different levels of representation. There is now an emerging imaging literature that strongly supports the very rapid influence of higher-level semantic and phonological knowledge upon visual and auditory word recognition. For example, in an ERP study of visual lexical decision (Hauk, Patterson, et al., 2006), initial activation in the region of left vOTC at 100 ms was driven by bigram frequency in a visual lexical decision task, but after strong activation in the left anterior temporal lobe at around 150 ms for words of low bigram frequency, the next surge of left vOTC activation patterned with lexicality at 200 ms, which was well before the behavioural response (see also Hauk, Coutout, Holden, & Chen, 2012). Turning to speech, Sohoglu, Peelle, Carlyon and Davis (2012) manipulated prior knowledge of the content of degraded speech using previously presented text, and found that this modulated EEG/MEG activity in the left inferior frontal gyrus as early as 90–130 ms, which was well before that seen in the superior temporal gyrus after 270 ms.

These results are very interesting because they indicate a role for feedback during word recognition in a way not permitted by consideration of behaviour alone. There is clearly very early activation of regions involved in semantic processing. The fact that these areas show sensitivity to lexicality/familiarity before more posterior form-based regions could be interpreted as supporting a view in which lexical representations reduce to top–down activation from semantic representations. However, it may also be that these form-based regions follow a different time course of activation, with initial sensitivity to subword properties shifting to a lexicality effect. This latter possibility could be accommodated by the distributed functional view where attractors act as lexical representations. It is very difficult to determine the extent to which early activation in one brain region causes a pattern of activation in another region, and consideration of the time course of unit activations in connectionist models could provide concrete predictions to test in future neuroimaging experiments.

## Development of lexical representations

Connectionist models, by definition, derive their representations via adjustments on initially random weights between processing units through a process of learning that involves exposure and usually error correction. As such, issues around learning of lexical representations may seem to map fairly directly onto the issue of distributed versus localist representations; however, this is not necessarily so. There are in fact models that include localist structural representations where the weights on connections between them are learnt (e.g., Dandurand, Hannagan, & Grainger, 2013; Mirman, McClelland, & Holt, 2006; Perry et al., 2007, 2010). When this is adopted as a theoretical standpoint, it can be considered ‘localist connectionism’ (Grainger & Jacobs, 1998); however, localist structural representations have also been adopted in the connectionist domain to render large-scale models tractable in terms of computational processing demands (Kello, 2006; Sibley et al., 2008).

Allowing the connections between localist representations to be learnt does not speak directly to their initial formation, yet understanding of this process is crucial if we are to accurately capture language development. A recent simulation allows for the formation of a localist lexical orthographic representation once initial translation of a letter string via subword orthography to phonology mappings makes contact with a pre-existing phonological lexical representation (Ziegler, Perry, & Zorzi, 2014). This process is a computational implementation of Share's (1995) self-teaching hypothesis, but it merely defers the problem of formation of a lexical entry from the orthographic to the phonological level – it does not tell us how the phonological lexical representations are formed. In contrast, in models that map directly from raw visual or auditory inputs, distributed lexical representations will emerge over the course of learning in the hidden units that mediate access to semantic and articulatory outputs via recurrent connections within and between layers (Chang et al., 2013; Plaut & Kello, 1999). The formation of distributed functional lexical representations therefore falls naturally from models that focus on mapping raw inputs to meaning and speech.

While connectionist models learn their representations, this does not mean that they necessarily do so in precisely the same way as children. Some connectionist models of reading development have captured this process well with a standard training regime (e.g., Harm & Seidenberg, 1999; Karaminis & Thomas, 2010; Ueno et al., 2011). Other models have used pre-training and more psychologically plausible training vocabularies and techniques, which have improved the fit of the models to children's data considerably (Hutzler, Ziegler, Perry, Wimmer, & Zorzi, 2004; Powell, Plaut, & Funnell, 2006). Clearly, developmental trajectories, like functional neuroanatomy, reflect a potential source of constraint upon model design that can be utilised in future. More generally, the fact that connectionist models can learn their representations allows them to speak to issues concerning not only normal and disordered language development (Harm, McCandliss, & Seidenberg, 2003; Harm & Seidenberg, 1999; Joanisse & Seidenberg, 2003), but also language function in progressive disorders (Rogers, Lambon Ralph, Garrard, et al., 2004; Woollams et al., 2007) and the possibility for relearning and rehabilitation after brain damage (Abel, Grande, Huber, Willmes, & Dell, 2005; Plaut, 1996; Welbourne & Lambon Ralph, 2005, 2007).

## Conclusions and future directions

This paper has considered a number of different computational implementations of lexical representations that seem warranted to account for the lexicality effects seen in visual and auditory language-processing tasks. Distributed functional representations were favoured by virtue of their efficiency and flexibility. Within connectionist models using such representations, one possibility is that lexical knowledge reduces to semantic activation. An alternative proposal outlined here is that whole word knowledge is also captured by a system of attractors at levels intermediate between form and meaning, formed during learning via recurrent connections between units within and across levels. Within this view, the nature of the stimuli and the demands of the task will determine where and when these functional lexical representations emerge, hence they are fluid. This latter proposal has the virtue of allowing a degree of modality and task specificity suggested by selective impairments in some neuropsychological patients and variability in the timing and location of lexicality effects in neuroimaging studies.

Further research is needed to tease apart the contributions of semantic versus form-based lexical knowledge to word recognition. This will require direct comparisons between lexicality effects and semantic effects, and also more parametric manipulations sensitive to graded specialisation. Comparisons over modalities and tasks are needed to determine the degree of specificity of lexical representations. Such research will preferably use neuroimaging techniques with sufficient temporal resolution to understand the flow of activation within the system prior to behavioural response. Simulation of these data will require models that start with approximations of raw visual and auditory inputs and incorporate at least some degree of recurrence between and within levels. Within such attractor networks, it is necessary to explore how the various possible performance metrics correspond to both behavioural and neuroimaging data.

In summary, this paper has highlighted that there are a variety of implementation options for lexical representations in connectionist models, both in terms of the nature of the semantic system and also for distributed functional representations as hidden unit attractors, ranging from discrete sets of hidden units for each modality/task to graded specialisation within a single integrative layer. Neuroimaging evidence can be used to constrain the range of possible implementations, but most current analysis techniques yield discrete cortical areas and are not sensitive to graded influences of form- and meaning-based variables. Neuroimaging evidence has its own limitations in terms of the cognitive interpretation of activation differences, and consideration of hidden unit dynamics in connectionist models with different architectures may shed light on the neural data. Ultimately, a full understanding of the source of lexicality effects in behaviour will require a dialogue between modelling and neuroscience through which they will converge on the precise form of lexical representations in the mind and brain.
